# Long-Term Follow-Up Outcomes after Percutaneous US/CT-Guided Radiofrequency Ablation for cT1a-b Renal Masses: Experience from Single High-Volume Referral Center

**DOI:** 10.3390/cancers12051183

**Published:** 2020-05-07

**Authors:** Giovanni Mauri, Francesco Alessandro Mistretta, Guido Bonomo, Nicola Camisassi, Andrea Conti, Paolo Della Vigna, Matteo Ferro, Stefano Luzzago, Daniele Maiettini, Gennaro Musi, Nicolò Piacentini, Gianluca Maria Varano, Ottavio de Cobelli, Franco Orsi

**Affiliations:** 1 Division of Interventional Radiology, IEO, European Institute of Oncology IRCCS, 20141 Milan, Italy; guido.bonomo@ieo.it (G.B.); nicola.camisassi@ieo.it (N.C.); paolo.dellavigna@ieo.it (P.D.V.); daniele.maiettini@ieo.it (D.M.); gianluca.varano@ieo.it (G.M.V.); franco.orsi@ieo.it (F.O.); 2 Department of Oncology and Hematology-Oncology, Università degli Studi di Milano, 20122 Milan, Italy; ottavio.decobelli@ieo.it; 3 Department of Urology, European Institute of Oncology IRCCS, 20141 Milan, Italy; FrancescoAlessandro.Mistretta@ieo.it (F.A.M.); andrea.conti@ieo.it (A.C.); matteo.ferro@ieo.it (M.F.); stefano.luzzago@ieo.it (S.L.); gennaro.musi@ieo.it (G.M.); dott.nicolo.piacentini@gmail.com (N.P.)

**Keywords:** renal cancer, image-guided thermal ablation, radiofrequency, survival, ultrasound, computed tomography

## Abstract

Image-guided thermal ablations are increasingly applied in the treatment of renal cancers, under the guidance of ultrasound (US) or computed tomography (CT). Sometimes, multiple ablations are needed. The aim of the present study was to evaluate the long-term results in patients with renal mass treated with radiofrequency ablation (RFA) with both US and CT, with a focus on the multiple ablations rate. 149 patients (median age 67 years) underwent RFA from January 2008 to June 2015. Median tumor diameter was 25 mm (IQR 17–32 mm). Median follow-up was 54 months (IQR 44–68). 27 (18.1%) patients received multiple successful ablations, due to incomplete ablation (10 patients), local tumor progression (8 patients), distant tumor progression (4 patients) or multiple tumor foci (5 patients), with a primary and secondary technical efficacy of 100%. Complications occurred in 13 (8.7%) patients (6 grade A, 5 grade C, 2 grade D). 24 patients died during follow-up, all for causes unrelated to renal cancer. In conclusion, thermal ablations with the guidance of US and CT are safe and effective in the treatment of renal tumors in the long-term period, with a low rate of patients requiring multiple treatments over the course of their disease.

## 1. Introduction

Kidney cancer accounts for approximately 3% and 5% of all new cancer cases in the United States in 2019, in females and in males respectively, with an estimated overall incidence of 73,820 new cases in 2019 [1]. The incidence of kidney cancer is increasing worldwide [2]. This can be partially explained by an increase in known risk factors, such as smoking or obesity, but also to the increasing incidental detection of small tumors during imaging exams performed for other reasons [2,3]. Radical or partial nephrectomy is still considered the first choice treatment [4], while image-guided thermal ablation has been proposed as an effective minimally invasive alternative, with reported good results and low complications [5,6,7,8]. Image-guided thermal ablations have been demonstrated to be particularly effective in the treatment of small kidney cancers < 4 cm [8,9].

Among different ablative techniques, radiofrequency ablation (RFA) has been one of the first applied in the treatment of kidney cancer [8,10,11]. Ultrasound (US), providing real time visualization, represents the most widely used technique for guiding ablations in the abdomen, particularly in Europe and Asia [12,13], while computed tomography (CT) is preferred in several centers, mainly in the United States [14,15]. Combination of US and a cross-sectional imaging modality such as CT might improve the visualization during treatment and improve results and safety of the procedure [16,17,18]. Thus, since 2008, at our Center we started performing RFA of T1a-b renal tumors in a dedicated operating room equipped with both US and CT guidance. Furthermore, one of the limitations of image-guided thermal ablation in comparison with more invasive surgical resections is the possible need of performing multiple ablations in the same patient to achieve a curative treatment, for the presence of incomplete ablation, local tumor recurrence or new foci in the same organ. 

The aim of the present study was to retrospectively evaluate the long-term results in patients with T1a-b renal masses treated with RFA with the guidance of both US and CT, with particular focus on the multiple ablations rate.

## 2. Results

### 2.1. General Characteristics of the Study Populations

Median age was 67 years and most of patients were male (65.8%). More than half of population was overweight (33.6%) or obese (20.8%). Median tumor diameter was 25 mm (IQR 17–32 mm) and most of patients had T1a lesions (87.2%), relative to T1b. A higher proportion of clear cell renal cell carcinoma (RCC) (45.6%) was identified, relative to papillary (8.1%), chromophobe (2.0%), and benign tumors (8.7%). Nevertheless, a high rate of not diagnostic biopsies (35.6%) was identified. Higher rates of right renal tumors (53.7%), as well as of lesions in proximity to renal sinus (26.2%) were identified. Higher rates of ≥ 50% intraparenchymal lesions (59.7%), as well as of PADUA score 8–12 (52.3%) were recorded. Patients’ characteristics are shown in Table 1.

After stratification according to age ≤70 vs. >70 years, among all descriptive covariates, a statistically significant difference was recorded for tumor diameter, which was higher for older patients (23 vs. 30 mm; *p* < 0.01), and for T-stage, with higher rate of T1b recorded in older patients (6.6 vs. 22.4%; *p* = 0.01). After stratification according to gender female vs. male, among all descriptive covariates, a statistically significant difference was recorded only for tumor deepness, with higher rate of ≥ 50% intraparenchymal lesions in women (72.5 vs. 53.1%; *p* = 0.03). After stratification according to BMI normal vs. overweight/obese, among all descriptive covariates, a statistically significant difference was recorded for tumor diameter, which was higher for overweight/obese patients (23 vs. 26 mm; *p* = 0.02), for T-stage, with higher rate of T1b recorded in overweight/obese patients (62.9 vs. 21.0%; *p* < 0.01), and for Padua score, with a higher rate of 8–12 points in overweight/obese patients (41.2 vs. 61.7%; *p* = 0.02), relative to normal weight patients. Patients’ characteristics after stratification are shown in Table 2.

### 2.2. Primary Endpoints

Overall median follow-up was 54 months (interquartile range [IQR]: 44–68). Of all included patients, 27 (18.1%) patients received multiple ablations, due to incomplete ablation (10 patients, 6.7% of all patients), or local tumor progression (8 patients, 5.4% of all patients), or distant tumor progression (4 patients, 2.7% of all patients) or multiple tumor foci at diagnosis (5, 5.4% of all patients). Distant tumor progression occurred in three cases in an adrenal gland, and in one case in the abdominal muscular tissue. After the secondary treatment, no need for successive treatments was recorded in any patients. No statistically significant differences were found in multiple ablations rate after stratification according to either ≤70 vs. >70 years (18.7 vs. 17.2%; *p* = 0.9), female vs. male (15.7 vs. 19.4%; *p* = 0.7) or normal vs. overweight/obese patients (16.2 vs. 19.8%; *p* = 0.7). At separate univariable logistic regression models (ULRMs) focusing on predictors of multiple ablations, tumor diameter (odd ratio [OR]: 1.04; CI 1.00–1.08; *p* = 0.04) was associated with higher multiple ablations rate. The higher rate of multiple ablations was identified for masses located at sinus proximity (13 patients, 48.1% of patients who underwent multiple ablations). Conversely, posterior renal face (OR: 0.24; CI 0.08–0.66; *p* = 0.01) and median pole (OR: 0.19; CI 0.05–0.60; *p* = 0.01) were associated with lower multiple ablations rate, relative to sinus proximity. No statistically significant differences were found when age, gender, BMI, histovariant, T-stage, tumor laterality, tumor deepness, and PADUA score were tested (Table 3).

### 2.3. Secondary Endpoints

**Figure 1:**
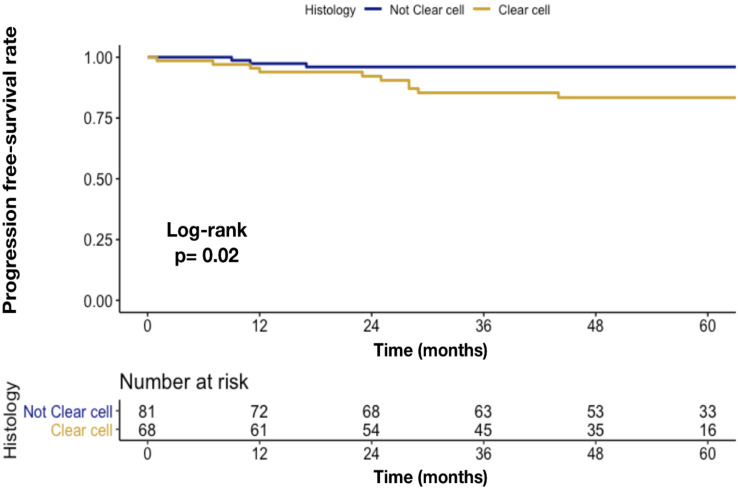
Kaplan-Meier plots depicting disease progression rate over time, according to histovariant clear cell RCC vs. not clear cell.

Image-guided thermal ablation was successfully performed in 139/149 patients (technical success 93.3%). In the incompletely ablated tumors, a second ablation was successfully performed, with a primary technical efficacy of 100%. Overall, disease progression was recorded in 12 (8.1%) patients. Of them, 8 (5.4%) patients had a local tumor progression, while 4 (2.7%) patients a distant progression. No statistically significant differences were found in disease progression rate after stratification according to either ≤70 vs. >70 years (9.9 vs. 6.9%; *p* = 0.7), female vs. male (7.8 vs. 9.2%; *p* = 1.0) or normal vs. overweight/obese patients (7.4 vs. 9.9%; *p* = 0.8). At Kaplan-Meier plots depicting local tumor progression rate over time, only clear cell RCC was associated with higher disease progression rate (17 vs. 4%; *p* = 0.02), relative to not clear cell RCC (Figure 1).

**Figure 2:**
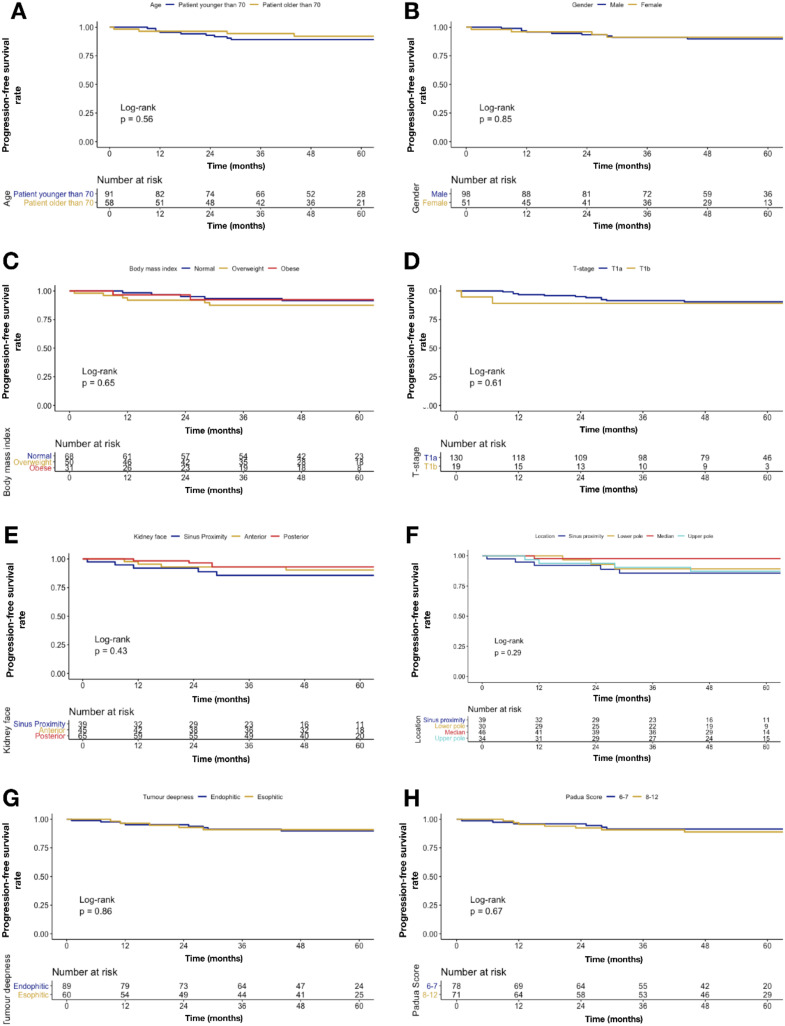
Kaplan–Meier plots depicting disease progression rate over time according to covariates: (**A**) age, (**B**) gender, (**C**) BMI, (**D**) T-stage, (**E**) renal face, (**F**) renal pole, (**G**) tumor deepness, and (**H**) PADUA score.

No statistically significant differences were found when age, gender, BMI, T-stage, renal face, renal pole, tumor deepness, and PADUA score were tested (Figure 2). 

All the 12 disease progressions detected during follow-up were successfully retreated with image-guided thermal ablations (secondary technical efficacy 100%).

Overall, at least one complication occurred in 13 (8.7%) patients. Specifically, after stratification according to SIR classification system, Grade A complication occurred in 6 (4.0%; small hematoma with no consequence), Grade C in 5 (3.4%; urinary leakage), and Grade D in 2 (1.3%; bleeding that required radiologic embolization) patients. No statistically significant differences were found in the complication rate after stratification according to either ≤70 vs. >70 years (8.8 vs. 8.6%; *p* = 1.0) or female vs. male (9.8 vs. 8.2%; *p* = 0.9), or normal vs. overweight/obese patients (5.9 vs. 11.1%; *p* = 0.4). Overall mortality rate was 16.1% (24 patients). All patients died due to causes not related to cancer.

## 3. Discussion

Image-guided thermal ablations have been initially introduced in the treatment of liver tumors, as a potential treatment of unresectable hepatocellular carcinomas [19,20,21] in the early 90s’. Nowadays, image-guided thermal ablations are considered the first choice treatment option for patients with small hepatocellular carcinomas [22]. In 1997, Zlotta et al., reported the first use of image-guided radiofrequency ablation in the treatment of a renal cancer [23], and nowadays this technique is included as a potential treatment in several guidelines by urological and radiological societies [17,24,25]. At the present, the majority of literature on the topic is based on small retrospective study, generally with short follow-up, and clinical series are based on US or CT guided ablations [8,9,10,11,12,13,14,15]. One of the key technical aspects to improve the results of ablation is the reliability of the image guidance. For this reason, in our center, we started to perform renal ablation in a dedicated setting, with both US and CT available, in order to merge the benefit of both techniques to improve the clinical results. The relevance of application of multiple imaging techniques to image-guided thermal ablation is underlined by the flourishing of experiences and papers on fusion imaging applied to thermal ablations in recent years [18,26,27,28,29]. Use of both US and CT during the ablative procedure, might also increase the ability to immediately detect a vital unablated residual tissue, and thus to guide an immediate intraprocedural second ablation. The possible occurrence of an incomplete ablation is among the drawbacks of image-guided thermal ablations, and can particularly occur in case of large or centrally located tumors [30,31,32,33]. However, one of the principal aims of application of image-guided thermal ablation is to lower the invasiveness of treatment in frail patients unsuitable for surgery, or to minimize renal function damage particularly when surgery would require total nephrectomy. In this setting, the need of multiple treatments is generally accepted and taken into account during multidisciplinary discussions when establishing the patient treatment strategy. In fact, with a second ablation complete tumor ablation can be achieved in the large majority of cases [30,31,34]. Furthermore, patients can have a local tumor progression during follow-up, develop new foci of renal tumors in the same or in the contralateral kidney, or distant metastases. In all these scenarios, image-guided thermal ablation can be applied to achieve disease control [30,33,34,35,36]. Thus, it might easily occur that a patient requires multiple ablations over the course of his disease. Our paper was thus mainly focused on analyzing long-term results in patients treated with the guidance of both US and CT, with particular focus on factors related to multiple treatments. In our series, with the guidance of both US and CT, we were able to achieve complete ablation in 93% of patients after one ablation, and with a second ablation to achieve a primary and secondary technical efficacy of 100%. Overall, 18% of the included patients required multiple ablations. Notably, the need of multiple ablations was significantly increased in larger tumors and in tumors that were centrally located. Furthermore, analyzing the local tumor progression rate, we found that only clear cell RCC was associated with higher local tumor progression rate. In our series, image-guided thermal ablation was confirmed to be a safe procedure, with an overall rate of complications of 8.7%, the majority being minor complications. The very low complications rate in our series underline the great potential of image-guided thermal ablation in the treatment of patients with renal tumor, particularly for those considered at high risk for a surgical treatment. No variable was found to be correlated with the complications rate, even if a tendency to a higher complication rate was found in overweight/obese patients.

Our results are comparable with previous literature. Wah T.M. et al. [8] reported results in a series of 200 patients treated with image-guided RFA, with a primary technical and overall technical success rate 95.5% and 98.5%, respectively. Similar to our results, lower success rate was found in large tumors and in tumors that were centrally located. In addition, in their series, major complications were associated with centrally located or lower pole tumors. In our series, no correlation was found between location and complications rate. This might be due to the regular application of retrograde pyeloperfusion to protect the collecting system in our center. Similarly, Zagoria et al., analyzing the results in 125 renal tumors treated with CT guided thermal ablations, found a complete ablation in 93% of cases, being the tumors smaller than 3.7 cm always completely ablated [15]. They found that, for each 1 cm increase in tumor diameter over 3.6 cm, the likelihood of tumor free survival decreased by a factor of 2.19. In a recent paper comparing results of image-guided thermal ablation with partial nephrectomy in an overall cohort of 1955 patients, Yu j. et al. [35] found no differences between local tumor progression, cancer-specific survival, and distant metastases in the two groups. Notably, they found smaller drop in estimated glomerular filtration rate at discharge, smaller estimated blood loss, lower cost, shorter operative time, and shorter postoperative hospitalization time in image-guided thermal ablation group in comparison with partial nephrectomy.

Our study has some limitations. First, it is a retrospective study, involving a large period of clinical practice. Thus, the considered series include patients treated at the very beginning of the experience, and clinical indications, technical equipment, and practice evolved over the time. Furthermore, this study represents the practice of a high volume oncological referral center, where all the technological facilities were available. Thus, our results might be difficult to be generalized. Even if considering a large period of time with a high number of enrolled patients, the low rate of events might have determined an impossibility of detecting some statistically significant correlations. Particularly, the low number of patients with disease progression should be taken into account when considering our results. Then, as we started our practice directly with using both US and CT for performing renal ablation, it is not possible to make a direct comparison with a group of patients treated with only US or only CT.

## 4. Materials and Methods

### 4.1. Study Population

Institutional review board approval was obtained (European Institute of Oncology, IRCCS, number of registration 2382), and patients’ informed consent was waived. From a prospectively collected database we retrospectively identified 149 patients affected by T1a-b renal mass and treated with RFA from January 2008 to June 2015. All patients received a image-guided biopsy before the treatment. Indication for ablation was always discussed by a multidisciplinary team involving urologists, nephrologists, surgeons, and interventional radiologists. Were included for treatment patients older than 18 years, with a biopsy-proven renal mass up to 7 cm or a new growing lesion in patients previously treated surgically for renal mass. Exclusion criteria included metastatic disease and coagulopathy.

### 4.2. RFA Procedure

All procedures were performed under general anesthesia, in a dedicated operating room equipped with both US and CT, with the patient lying in the most favorable position for direct needle approach. All procedures were performed by a team of two interventional radiologists, with at least one with more than 10 years of experience. Adjunct procedures such as hydrodissection or pyeloperfusion were used when adequate upon team clinical judgment [6]. A preliminary US evaluation was used to establish the ideal path to reach the target lesion, and to establish the patient decubitus. Then, a contrast-enhanced CT (CECT) was performed to better visualize the lesion and surrounding anatomical structures. RFA was performed with an RF 3000 system (Boston Scientific, Natick, Massachusetts, MA, USA) and a retrievable hook-umbrella needle. The umbrella diameter was chosen case by case according the shape and size of the lesion (range 2–5 cm). The electrode was advanced under US guidance and repeated focused CT acquisitions were performed to confirm correct needle positioning. Ablation was performed with a dedicated protocol, increasing the power until roll-off was reached, twice per each needle deployment. At the end of the procedure, a CECT was performed for early treatment evaluation and to identify immediate complications. Patients underwent overnight observation, and were discharged the following day if clinically stable. Prior to discharge, a complete blood count and CECT were performed to rule out any occult complication.

### 4.3. Endpoints and Variables of Interest

Primary endpoint of the study was the multiple ablations rate (due to incomplete ablation, local tumor progression, or new foci).

Covariables consisted of: age, gender, body mass index ((BMI) continuously coded or normal vs. overweight vs. obese), tumor diameter (millimeters), T-stage (T1a or T1b), histovariant (clear cell, papillary, chromophobe, benign, or not diagnostic), tumor laterality (right, left or bilateral), tumor location (anterior lower pole, anterior median, anterior upper pole, posterior lower pole, posterior median, posterior upper pole, or sinus proximity), renal face location (anterior, posterior, sinus proximity), renal polar location (lower, median, upper, or sinus proximity), tumor deepness (≥50% intraparenchymal vs. <50% intraparenchymal), Padua score (6–7 vs. 8–12).

Secondary endpoints were technical success, primary and secondary technical efficacy rates, disease progression (local tumor progression, new foci, or distant metastases), and complication rate, defined according to standard criteria [37], and overall mortality (OM) rate. Particularly, ‘technical success’ describes whether tumor was treated according to the predetermined protocol, ‘primary technical efficacy’ weather target tumor was successfully ablated following the initial procedure, ‘secondary technical efficacy’ weather target tumor successfully underwent a ‘second ablation’ after identification of ‘residual unablated tumor’. ‘Local tumor progression’ is defined as the appearance of tumor foci at the edge of a previously completely ablated zone. ‘New foci’ are defined as new tumors in the same organ, to be differentiated from ‘distant metastases’ occurring in other organs. ‘Retreatment’ is the treatment of a local tumor progression, which is different from ‘second ablation’, performed for incomplete ablation. In order to identify all the patients that in the course of their disease underwent more than one ablation, we grouped together patients who underwent a second ablation for incompletely ablated tumor, a retreatment for a local tumor progression, or a new ablation for a new tumor foci in the group of patients with ‘multiple ablations’.

### 4.4. Statistical Analyses 

First, differences in descriptive characteristics were investigated after stratification according to age (≤70 vs. >70 years), gender (male vs. female) and BMI (normal weight vs. overweight/obese). In this step Chi-squared, Wilcoxon, or Mann-Whitney tests estimated difference in proportions or medians.

Second, ULRMs tested for predictors of multiple ablations. Specifically, separate ULRMs tested association between age, gender, BMI, tumor diameter, T-stage, renal face location, renal polar location, tumor laterality, tumor deepness, Padua score, histovariant, and retreatment.

Third, Kaplan-Meier (KM) plots depicted differences in disease progression (either local or distant disease progression) rate. All statistical tests were two-sided with a level of significance set at *p* < 0.05. Analyses were performed using the R software environment for statistical computing and graphics (version3.4.1; http://www.r-project.org/).

## 5. Conclusions

In conclusion, thermal ablation with the guidance of US and CT is safe and effective in the treatment of T1a-b renal tumors in the long-term period, with a low rate of patients requiring multiple treatments over the course of their disease.

## Figures and Tables

**Table 1:** Descriptive characteristics of 149 patients with non-metastatic T1a-b renal tumor treated with RFA from 2004 to 2015.

Characteristic		Overall (*n* 149)
Age	Median	67
	Interquartile Range	60–75
Gender (*n*; (%))	Male	98 (65.8)
	Female	51 (34.2)
Body mass index categories (*n*; (%))	Normal	68 (45.6)
	Overweight	50 (33.6)
	Obese	31 (20.8)
Tumor diameter (mm)	Median	25
	Interquartile Range	17–32
T-stage (*n*; (%))	T1a	130 (87.2)
	T1b	19 (12.8)
Histovariant (*n*; (%))	Clear cell	68 (45.6)
	Papillary	12 (8.1)
	Benign	13 (8.7)
	Chromophobe	3 (2.0)
	Not diagnostic	53 (35.6)
Tumor laterality (*n*; (%))	Right	80 (53.7)
	Left	66 (44.3)
	Bilateral	3 (2.0)
Tumor location (*n*; (%))	Anterior lower pole	9 (6)
	Anterior median	17 (11.4)
	Anterior upper pole	19 (12.8)
	Posterior lower pole	21 (14.1)
	Posterior median	29 (19.5)
	Posterior upper pole	15 (10.1)
	Sinus proximity	39 (26.2)
Tumor deepness (*n*; (%))	≥50% intraparenchymal	89 (59.7)
	<50% intraparenchymal	60 (40.3)
Padua score	6-7	71 (47.7)
	8-12	78 (52.3)
Incomplete ablation (*n*; (%))	Occurred	10 (6.7)
Local tumor progression (*n*; (%))	Occurred	8 (5.4)
Distant tumor progression (*n*; (%))	Occurred	4 (2.7)
Retreatment rate (*n*; (%))	Retreated	27 (18.1)
Complication	A	6 (4.0)
	B	0 (0)
	C	5 (3.4)
	D	2 (1.3)
	No complications	136 (91.3)
Overall mortality rate (*n*; (%))	Occurred	24 (16.1)

**Table 2:** Descriptive characteristics of 149 patients with non-metastatic T1a-b renal tumor treated with RFA from 2004 to 2015, stratified according to age group (younger or equal to 70 years vs. older than 70 years), gender (female vs. male), and BMI (normal vs. overweight/obese). Covariates consisted of: age, gender, BMI, tumor diameter, tumor stage, histovariant, tumor location, tumor laterality, tumor deepness, and PADUA score.

Characteristics		Overall (*n* 149)	≤ 70 Years (*n* 91; 61.1%)	> 70 Years (*n* 58; 38.9%)	*p* Value
Tumor diameter	Median	25	23	30	<0.01
	Interquartile Range	17–32	15–28	20–38	
T-stage (*n*; (%))	T1a	130 (87.2)	85 (93.4)	45 (77.6)	0.01
	T1b	19 (12.8)	6 (6.6)	13 (22.4)	
**Characteristics**		**Overall** **(*n* 149)**	**Female** **(*n* 51; 34.2%)**	**Male** **(*n* 98; 65.8%)**	***p* Value**
Tumor deepness (*n*; (%))	≥50% intraparenchymal	89 (59.7)	37 (72.5)	52 (53.1)	0.03
	<50% intraparenchymal	60 (40.3)	14 (27.5)	46 (46.9)	
**Characteristics**		**Overall** **(*n* 149)**	**Normal** **(*n* 69; 45.6%)**	**Overweight/obese** **(*n* 81; 54.4%)**	***p* Value**
Tumor diameter	Median	25	23	26	0.02
	Interquartile Range	17–32	16–30	17–38	
T-stage (*n*; (%))	T1a	130 (87.2)	66 (97.1)	64 (79.0)	<0.01
	T1b	19 (12.8)	2 (2.9)	17 (21.0)	
Padua score	6–7	71 (47.7)	40 (58.8)	31 (38.3)	0.02
	8–12	78 (52.3)	28 (41.2)	50 (61.7)	

**Table 3:** Univariable logistic regression models prediction of multiple ablations rate according to clinical and pathological characteristics.

Characteristics	Characteristics	Univariable Odds Ratio	2.5%	97.5%	*p*-Value
Tumor diameter	Continuously coded	1.04	1.00	1.08	**0.04**
T-stage	T1a	Ref.	Ref.	Ref.	Ref.
	T1b	1.75	0.52	5.13	0.3
Renal face location	Sinus proximity	Ref.	Ref.	Ref.	Ref.
	Anterior	0.37	0.12	1.02	0.1
	Posterior	0.24	0.08	0.66	**0.01**
Renal polar location	Sinus proximity	Ref.	Ref.	Ref.	Ref.
	Lower	0.40	0.11	1.23	0.1
	Median	0.19	0.05	0.60	**0.01**
	Upper	0.34	0.10	1.05	0.1
Tumor laterality	Right	Ref.	Ref.	Ref.	Ref.
	Left	1.39	0.58	3.33	0.5
	Bilateral	11.33	1.01	255.53	0.054
Tumor deepness	≥50% intraparenchymal	Ref.	Ref.	Ref.	Ref.
	<50% intraparenchymal	0.46	0.17	1.11	0.1
Padua Score	8–12	Ref.	Ref.	Ref.	Ref.
	6–7	0.59	0.24	1.38	0.2
Histovariant	Not clear cell	Ref.	Ref.	Ref.	Ref.
	Clear cell	1.96	0.85	4.68	0.1
Gender	Male	Ref.	Ref.	Ref.	Ref.
	Female	0.77	0.30	1.86	0.6
Age	Continuously coded	1.02	0.98	1.06	0.4
Body mass index	Continuously coded	1.02	0.94	1.09	0.6

Ref = reference.
